# Mapping the research of nursing in Parkinson’s disease: a bibliometric and quantitative analysis

**DOI:** 10.3389/fneur.2024.1412158

**Published:** 2024-09-25

**Authors:** Han-Bing Liao, Yan-Lin Fang, Shu-Yi Chen, Yu-Shan Yin, Jiao Li, Peng Zhou, Bin Li, Xing-Zuan Jiang, Ying-Fang Lei

**Affiliations:** ^1^Department of Geriatrics, The Second Affiliated Hospital of Guangzhou Medical University, Guangzhou, China; ^2^Department of Neurology, Institute of Neuroscience, Key Laboratory of Neurogenetics and Channelopathies of Guangdong Province and the Ministry of Education of China, The Second Affiliated Hospital, Guangzhou Medical University, Guangzhou, China

**Keywords:** Parkinson’s disease, nursing care, bibliometric analysis, quality of life, dementia

## Abstract

**Background:**

Parkinson’s disease (PD) is a chronic and progressive neurodegenerative disorder. Clinically, the therapeutic strategy of PD could only alleviate the symptoms. Nursing plays a crucial role in providing patient education, symptom management, and psychosocial support. This study aims to analyze the current state and prospects of research in the field of Parkinson’s disease (PD) and its associated nursing care through bibliometric methods to explore the trends that May guide its future development.

**Methods:**

Literature related to Parkinson’s disease and nursing care was systematically searched by the Web of Science database from 1991 to 2023. Quantitative analysis of cooperative networks was conducted using bibliometric tools VOSviewer and CiteSpace.

**Results:**

The analysis covered 2,649 publications in the field of PD and nursing care, authored by 12,576 researchers from 3,869 institutions across 94 countries. The number of articles has steadily increased over the past 20 years. In this research field, the United States and the United Kingdom emerged as leading countries, and Radboud Universiteit Nijmegen was positioned as an international hub. *Movement Disorders* was identified as the journal with the highest output and with the most co-citation. Prof. Bastiaan R. Bloem published the most papers in the area, and Prof. Per Odin had the highest average citation. The major fields of these publications are clinical neurology, geriatrics & gerontology, multidisciplinary sciences, and health care sciences & services. Hot topics in the field predominantly revolve around Parkinson’s disease, quality of life, and dementia.

**Conclusion:**

Research in Parkinson’s disease and nursing care is experiencing a period of rapid growth, with continuous expansion in research scope and depth of investigation. One of the trends identified is the increasing focus on quality of life and the management of dementia in PD patients, reflecting the importance of these areas in research. The study further suggests that future advancements in the field May rely significantly on strengthening international collaborations and addressing global disparities in resource distribution, particularly by promoting research inclusivity and cooperation among low-resource countries.

## 1 Introduction

Parkinson’s disease (PD) is a chronic and progressive neurodegenerative disorder that affects millions worldwide (1, 2). It is characterized by the loss of dopaminergic neurons in the substantia nigra pars compacta and the presence of Lewy bodies in the brain (3). PD manifests with motor symptoms such as tremors, bradykinesia, and rigidity, as well as non-motor symptoms including depression, anxiety, and cognitive decline (4, 5). PD is one of the common neurological diseases causing a huge hurt to the physical and psychological suffering of patients. With the development of disease, the patients will gradually lose their ability to work and daily life. Clinically, the therapeutic strategy of PD is limited and could only alleviate the symptoms (6, 7). The management of PD requires a multidisciplinary approach, in which nursing plays a crucial role in providing patient education, symptom management, and psychosocial support (8).

The importance of nursing research in PD is well recognized, as effective nursing interventions can significantly improve the quality of life of individuals living with the condition. Nurses are often the longest-serving healthcare professionals in the lives of individuals with PD, providing consistent and reliable care throughout the trajectory of the disease. However, the nursing research field in PD is vast and complex, with numerous studies published each year across multiple journals (9). To gain a comprehensive understanding of the current state of nursing research in PD, a bibliometric analysis is a useful tool. Bibliometric analysis is a quantitative approach that involves the use of mathematical and statistical methods to study the patterns and relationships in the production and utilization of literature. It allows for the identification of research trends, hotspots, and emerging areas within a specific field (10, 11).

In this study, we conducted a bibliometric analysis to map the research trends and hotspots of nursing research in PD. The objectives of this analysis were to identify the main research topics and trends in nursing research on PD, identify the main contributors to this research, and explore the international collaboration in this field. We also aimed to identify potential gaps in the literature that require further exploration.

## 2 Materials and methods

### 2.1 Data retrieval and collection

This article retrieves the relevant literature on nursing study in PD from the Web Science Core Collection database (WoSCC). The search formula is: (((AB = (Parkinson’s disease)) OR AB = (paralysis agitans)) AND AB = (nursing OR Patient care OR Medical care)) OR (((TI = (Parkinson disease)) OR TI = (paralysis agitans)) AND TI = (nursing OR Patient care OR Medical care)). The search period is from 1991 to 2023, including all types of documents in this interval. The language is limited to English. The retrieval and extraction of data were performed by two independent researchers to avoid potential bias and improve the reliability of the results. The studies involving Parkinson’s disease and nurse care were included, while those published informally or not published in English were excluded.

### 2.2 Data analysis and visualization

This study mainly used VOSviewer 1.6.19 and CiteSpace 6.2.R4 to analyze the relevant literature. Firstly, VOSviewer was used to build a thesaurus of synonyms and merge the synonyms in the literature (12–14). Subsequently, CiteSpace was used to process the data downloaded from WOSCC and create a new project for visualization analysis (15–17). The period was set from 1991 to 2023, and each time slice was set to 1. When analyzing, individual node types were selected, and the threshold was set to (top N per slice) = 50, top N% = 20%. Next, visualization analysis was conducted on key information such as authors, countries/regions, journals, and institutions of these studies.

## 3 Results

### 3.1 Overview of included literature

Following the above retrieval strategy, a total of 2,649 pieces of literature are obtained for WOSCC. These literature were written by 12,576 authors from 3,869 institutions of 94 countries and cited 78,761 papers from 15,161 journals.

### 3.2 Annual publications and trends

**Figure 1:**
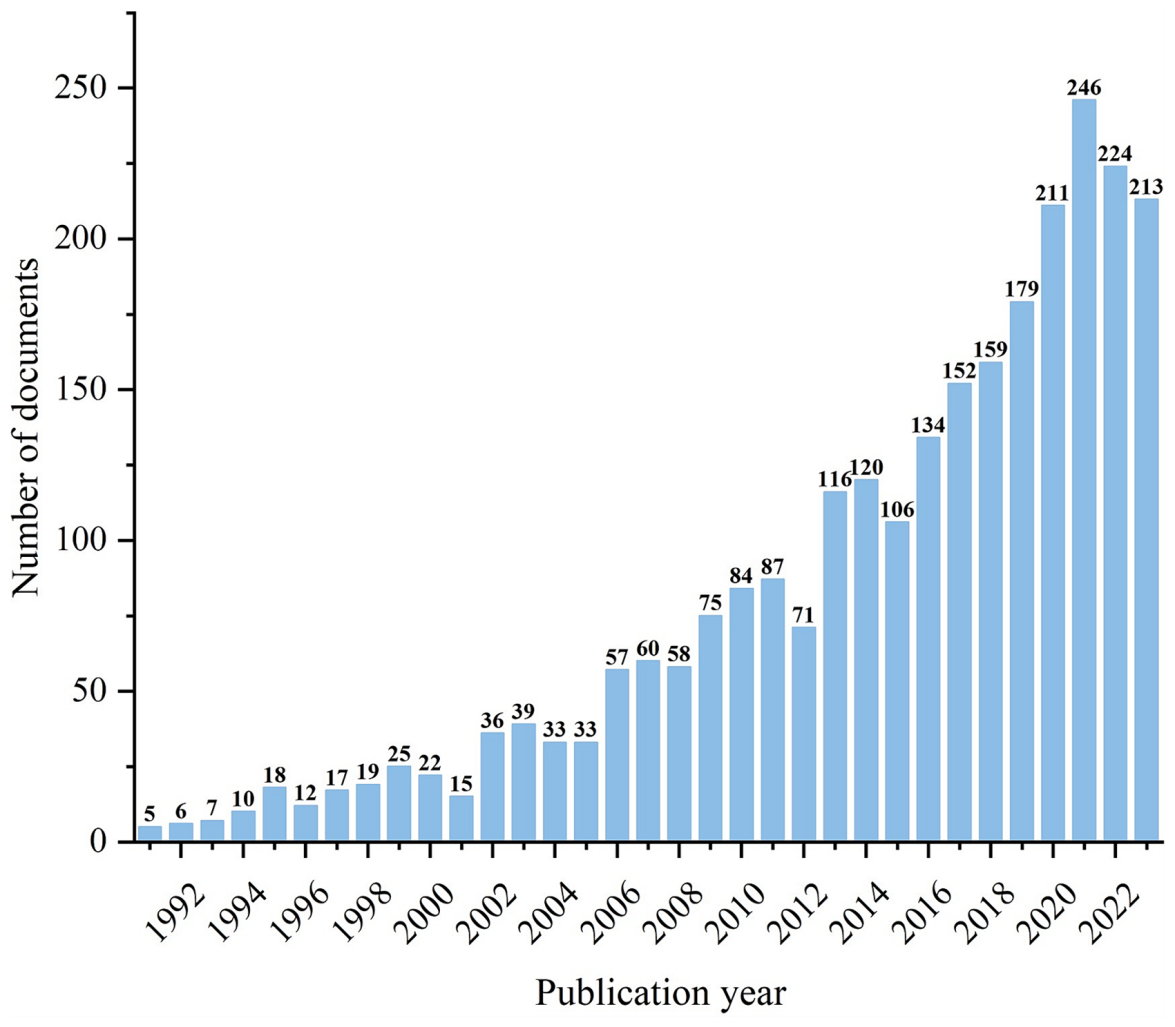
Analysis of the publication years of research literature related to PD and nursing.

The total number of papers published in this field has shown an increasing trend over time (Figure 1). After 2005, the number of related studies has increased exponentially, with the highest number of publications in 2021, indicating that this field has attracted increasing attention from scholars in recent years.

### 3.3 Author influence analysis

Bibliometrics analysis of authors in the field helps in identifying representative scholars and core research forces in the field (18, 19). The 2,903 authors in this field were analyzed by VOSviewer. The top five authors in terms of publication output are listed in Supplementary Table S1. The author with the highest number of publications and citations is Prof. Bastiaan R. Bloem from Radboud Universiteit Nijmege. The author with the highest average citation is Prof. Per Odin from Radboud Universiteit Nijmegen. These results indicate that these authors have a high influence in the field of PD and nursing.

**Figure 2:**
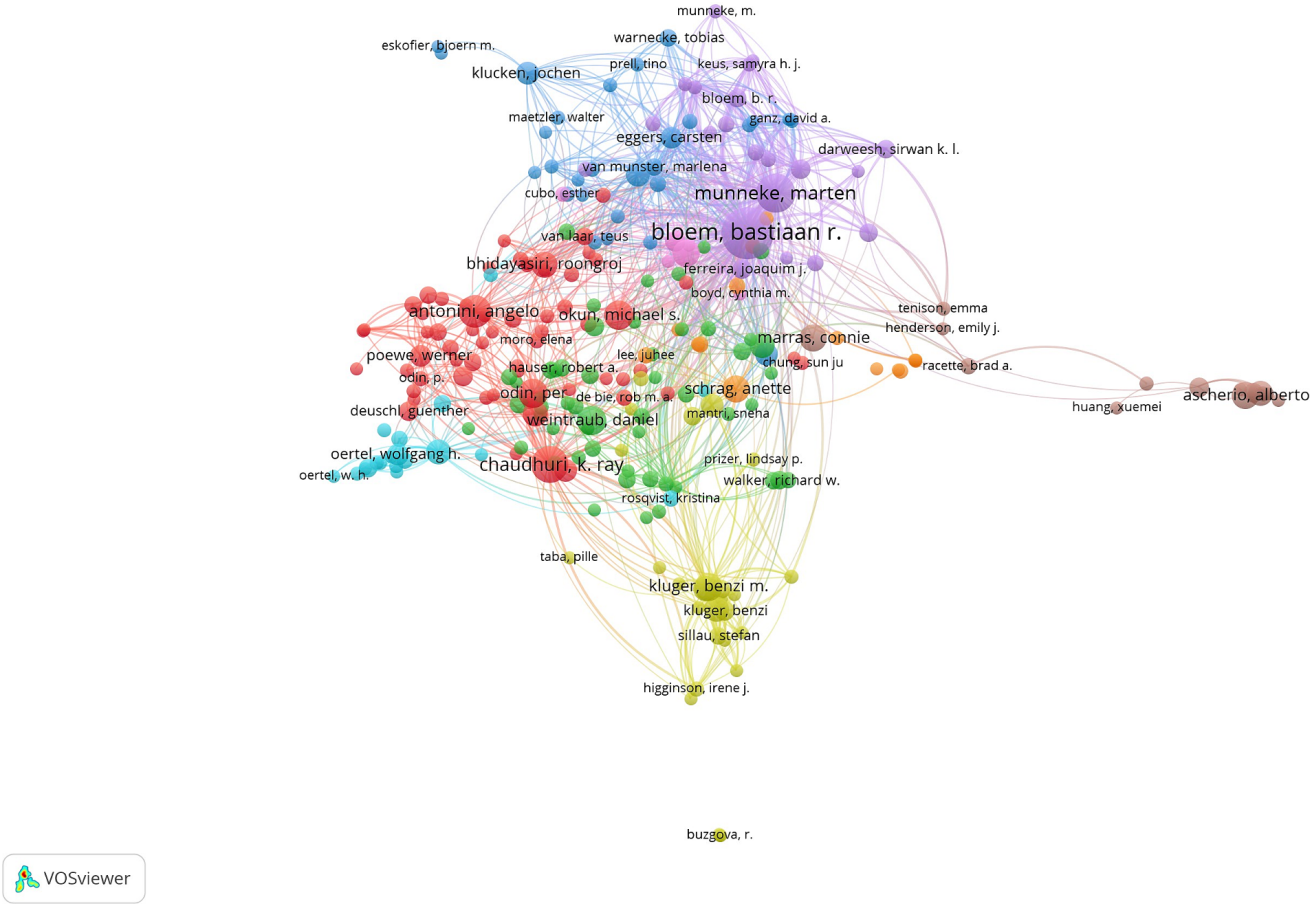
Author collaboration network for research related to Parkinson’s and nursing. The color of the nodes indicates different clusters; the size of the nodes denotes the number of documents output, with larger nodes signifying a greater volume of documents; the thickness of the lines represents the strength of collaboration, with thicker lines indicating tighter connections between two authors.

The authors who have published at least five articles were filtered to generate a co-authorship network map in the field, revealing information on representative scholars and their collaborations. It is evident from the map that there is a close connection among authors, with Bastiaan R. Bloem and Marten Munneke PT presenting the highest number of links (Figure 2).

### 3.4 Contributions of countries/regions

**Figure 3:**
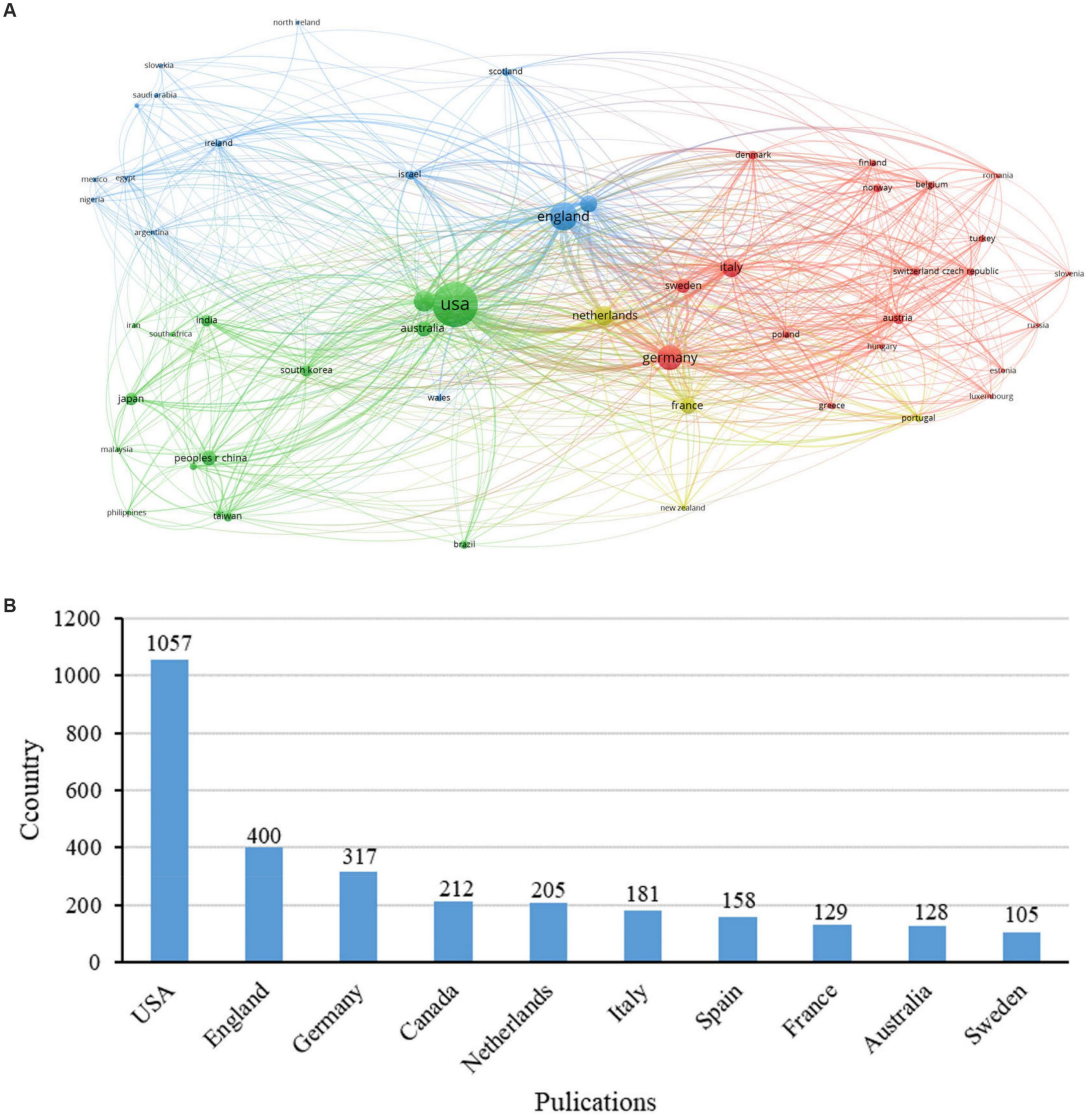
Schematic illustration of analysis of countries/regions. **(A)** Countries collaboration map; **(B)** Publication volume by regions in research on PD and nursing.

The contributions of nursing research in PD were accomplished by 52 countries. The majority of publications (83%, 2,191 papers) originated from the five top-ranking countries in this domain. To elucidate the contributions of individual countries more clearly, we employed the VOSviewer tool and visual analysis on countries with five or more publications, the findings of which are depicted in Figure 3A. A compilation of the top ten prolific publishing countries has been presented in Supplementary Table S2. A considerable disparity of publication across countries was presented. The United States was the country with the highest number of published articles, far outpacing other countries as illustrated in Figure 3B. The list is dominated by developed countries, indicating a discrepancy in the level of emphasis placed on this research area by various nations. The average number of publications from low-and middle-income countries in Latin America, Africa, and Asia was 9.84, significantly lower than that of high-income countries. An extensive collaborative network exists between the leading countries, including the United Kingdom, the United States, Germany, and France, while collaboration between low-and middle-income countries and developed countries is not very close (Figure 3B).

### 3.5 Institutional influence analysis

**Figure 4:**
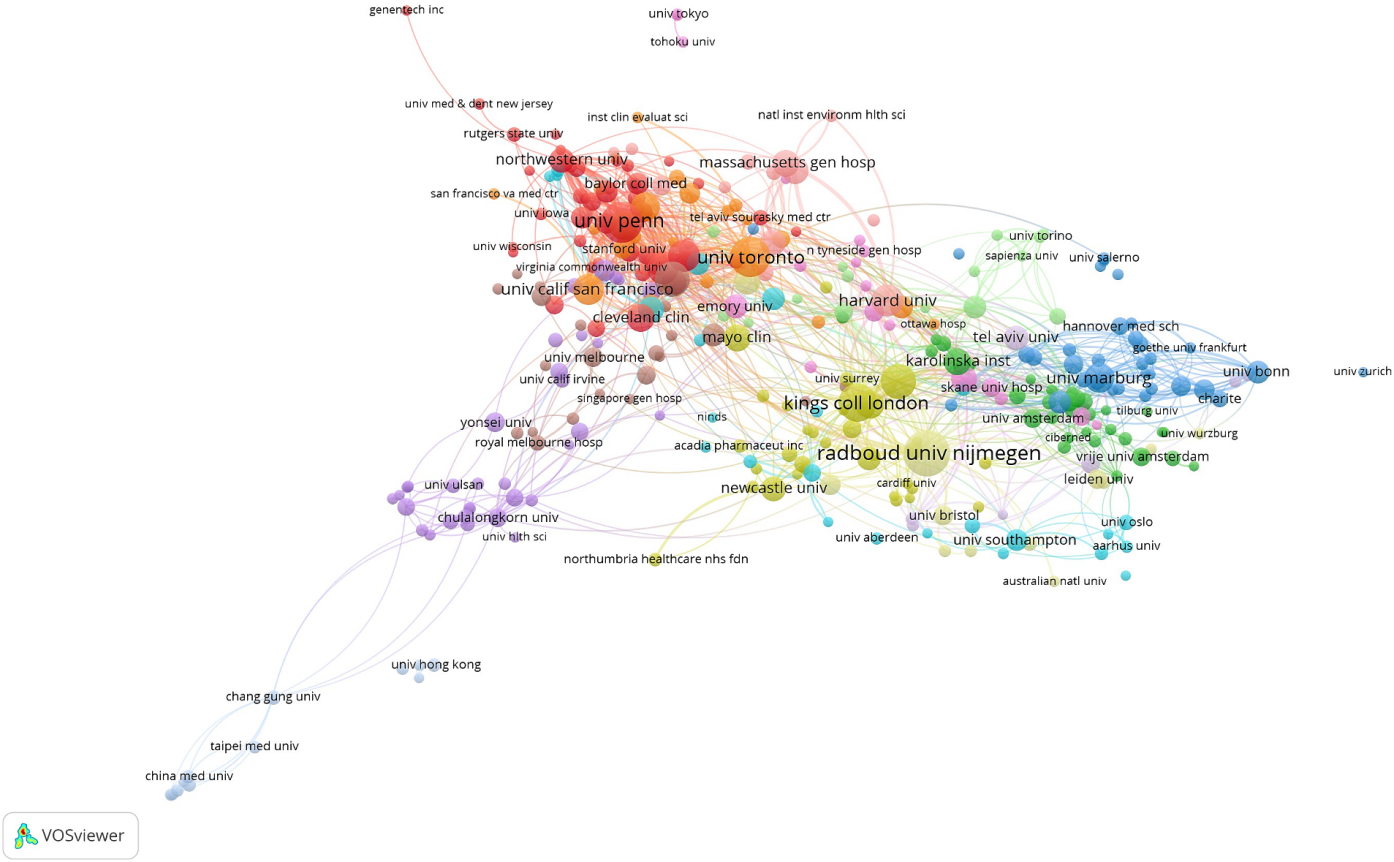
Collaboration network of institutions related to PD and nursing research.

So far, a total of 3,869 institutions have published literature in this field. Supplementary Table S2 presents the top ten institutions in terms of publication output. It can be observed that the institution with the highest number of publications is Radboud Universiteit Nijmegen (99 papers, 3.74% of the total), followed by the University of Pennsylvania (81 papers, 3.06% of the total) and the University of Toronto (75 papers, 2.83% of the total). From Supplementary Table S2 and Figure 4, it can be found that the publication output of leading research institutions accounts for a small proportion of the overall publication output, and a head effect has not yet been formed (20).

### 3.6 Journal analysis for articles published in the field

The 2,649 papers in this field were published in 872 journals. The top ten journals have contributed 728 papers on the subject, accounting for 27.48% of this field. “*Movement Disorders*” (IF: 8.6, Q1) stands as the journal with the highest output, publishing 234 articles. According to JCR 2022 standards, the majority of the journals within the top ten are categorized as either Q1 or Q2, with “*The Lancet Neurology*” (IF: 48, Q1) being identified as the journal with the highest impact factor, featuring 17 articles that garner an average of 96 citations each. Notably, both aforementioned journals are prominent within the clinical neurology sector, signifying an escalating interest in PD and nursing research among professionals in this discipline.

### 3.7 Analysis of co-cited journals

**Figure 5:**
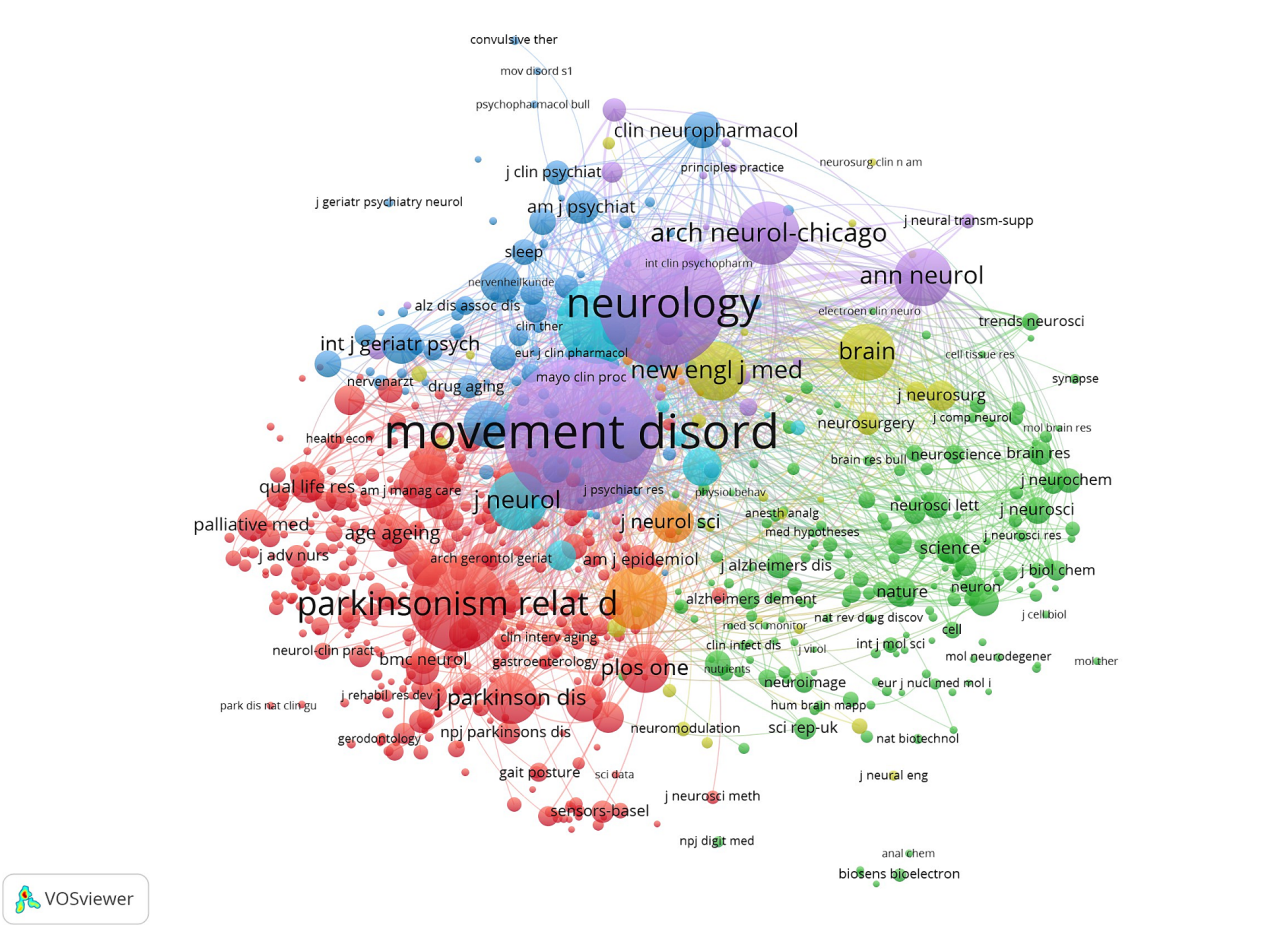
Co-cited map of journals in collaborative research related to Parkinson’s and nursing.

As shown in Supplementary Table S4, the journal with the highest citation frequency is “*Movement Disorders*” (9,947 citations), followed by “*Neurology*” (6,816 citations) and “*Parkinsonism & related disorders*” (3,825 citations). Among the top 10 journals with the highest citation frequency, the proportion of Q1 journals is the highest, indicating that articles in this field have a high level of influence. Using VOSviewer software, 735 journals with a minimum of 20 citations were analyzed. The network is mainly divided into four main clusters: Clinical Neurology, Geriatrics & Gerontology, Multidisciplinary Sciences, and Health Care Sciences & Services (Figure 5).

### 3.8 Co-cited reference analysis

**Figure 6:**
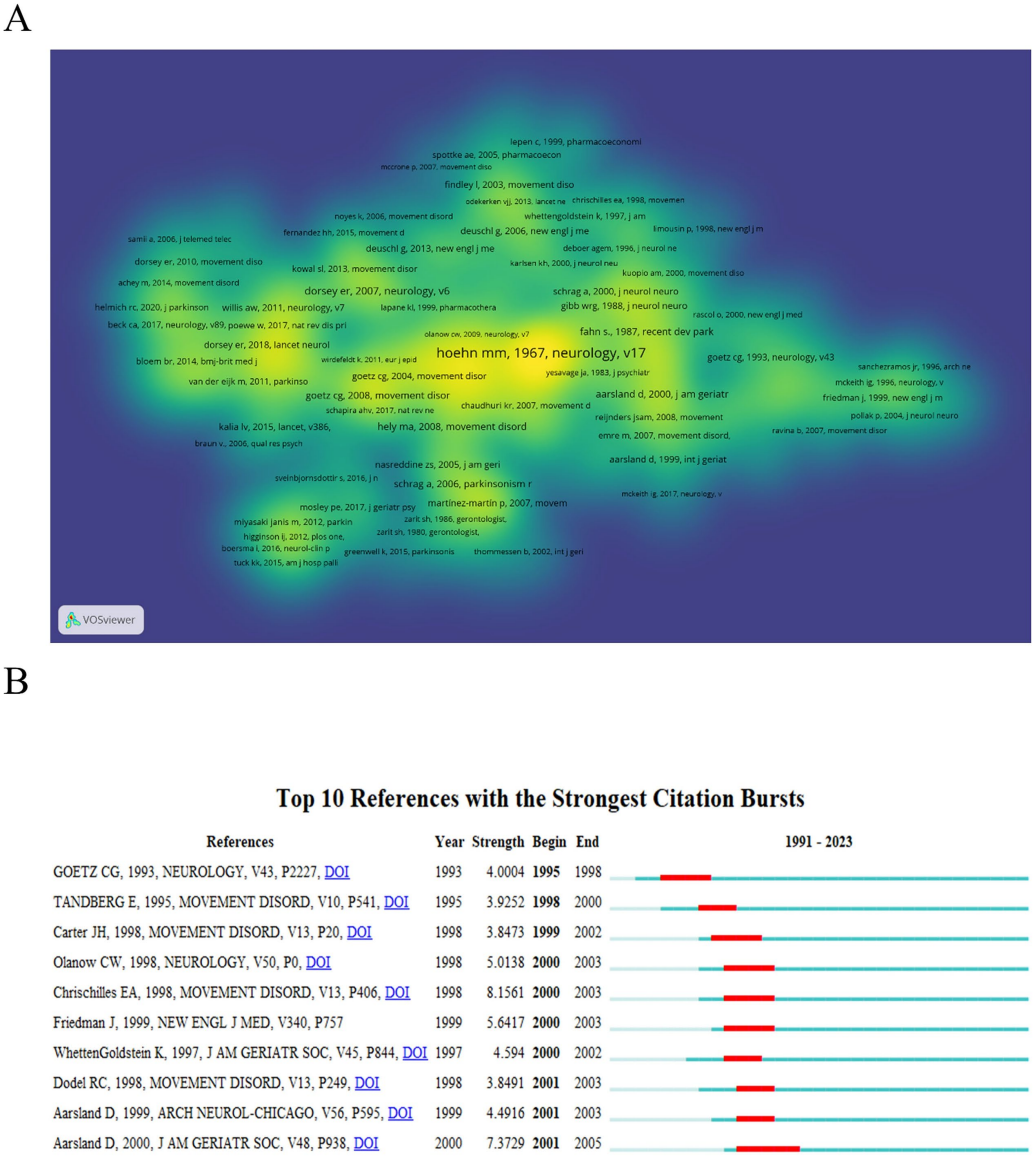
Schematic illustration of analysis of co-cited references. **(A)** The density visualization map of co-cited references based on VOSviewer. The color closer to yellow indicates a higher influence, while blue indicates a lower influence. **(B)** Burst strength and time duration of the top 10 references with the strongest citation bursts.

There are a total of 78,761 cited references in the field. *The Parkinsonism: onset, progression, and mortality* (21) has the highest number of citations, reaching 331. Figure 6A displays a visualization of the Co-cited density landscape, where areas closer to yellow indicate a higher influence of the literature, and those closer to blue suggest a lower influence. Figure 6B presents the top 10 co-cited references with the strongest citation bursts, with burst strength values ranging from 3.85 to 8.16. The literature with the highest burst strength (8.16) is *The health burdens of Parkinson’s disease* (22).

### 3.9 Analysis of keyword

**Figure 7:**
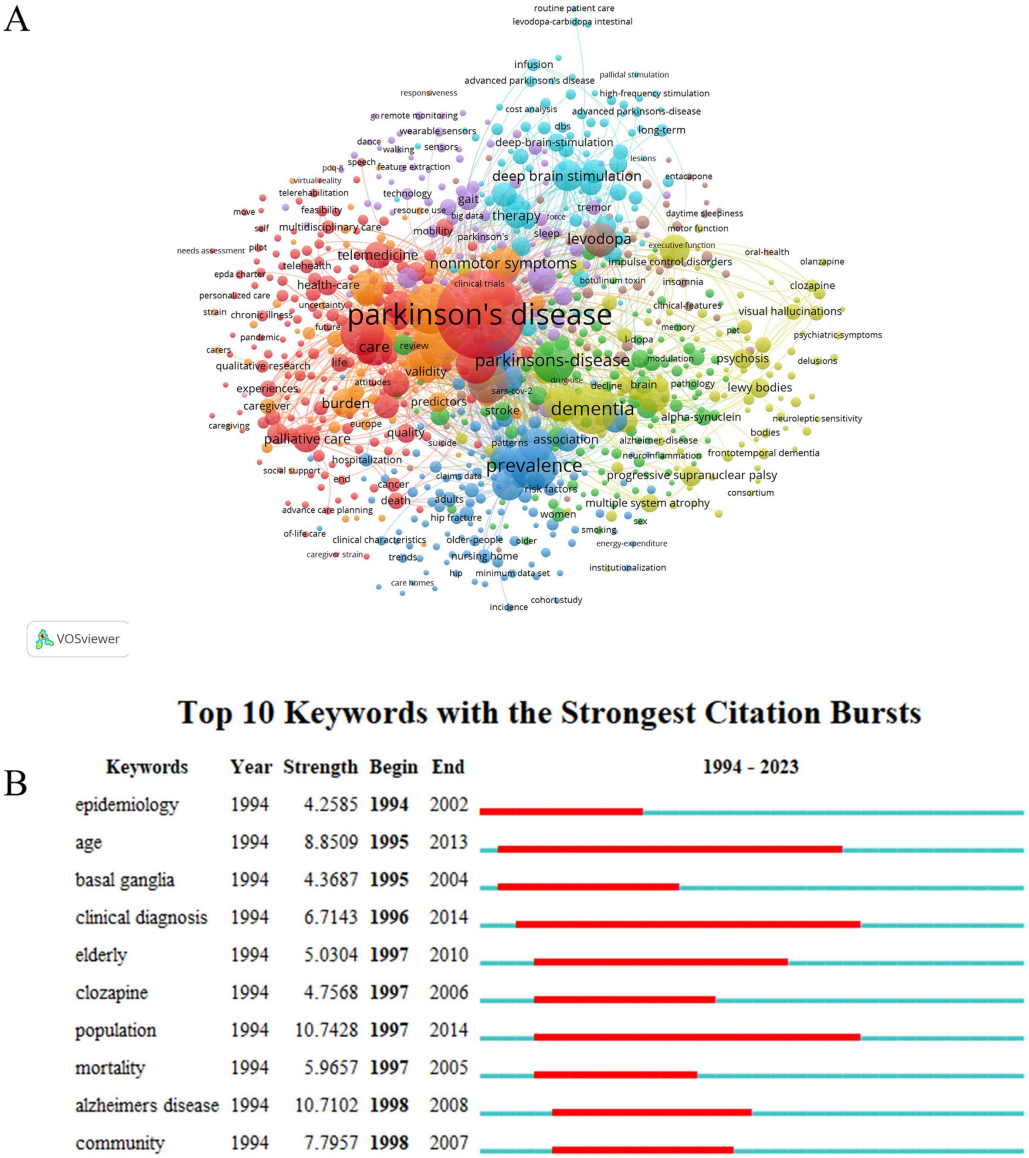
Schematic illustration of analysis of keywords. **(A)** Visualization map of keywords in PD and nursing research; **(B)** Burst strength and time duration of the top 10 keywords with the strongest citation bursts.

A keyword co-occurrence network view was constructed by utilizing VOSviewer, based on the analysis of 2,649 publications, to unveil the research hotspots and core concepts of a field (23). A total of 837 keywords that occurred at least five times were included for analysis. The top ten most frequent keywords were Parkinson’s disease, quality-of-life, dementia, prevalence, people, depression, symptoms, quality of life, nonmotor symptoms, levodopa, and risk (Figure 7A; Supplementary Table S6). Figure 7B displays the top ten keywords with the highest burst strength, with the burst strength values ranging from 4.28 to 10.71. Notably, the two keywords with the highest burst are “population” (10.74) and “Alzheimer’s disease” (10.71). Both the figure and table indicate that high-frequency keywords such as “Parkinson’s disease”, “quality-of-life”, “dementia”, “prevalence”, “people”, etc., form the representative academic terminologies of the field and denote its research hotspots.

## 4 Discussion

This study performed a bibliometric analysis on 2,649 publications related to PD care. Analysis indicated a steady annual growth rate of 14.6% since 1991, reflecting an increased volume of literature and heightened research activity in the field. This trend is likely associated with global population aging and lifestyle changes, leading to a rise in the incidence of PD and a correspondingly greater focus on its care and treatment research. Advances in medical technology and innovations in research methodologies are also identified as key drivers of scientific progress (24). Previous studies in this field focus on understanding and management of PD disease and improving the quality of life for individuals with PD. Future studies should aim to explore the impact of these trends on clinical practice and to identify potential areas for further research in PD care. This study provides a valuable resource for researchers, clinicians, and policy-makers in the field of PD nursing, and would be helpful in guiding future research directions and enhancing understanding of current research trends and hotspots.

Analysis of the contributions of different countries/regions revealed that publications of nursing research in PD are predominantly concentrated in developed countries. The United Kingdom, with the majority of top institutions, led international collaborations. Studies on PD were weaker in lower-middle income countries, in which PD would be less common due to a low average lifetime. It was indicated that the international distribution of this field was imbalanced. The developed countries May have high investment in healthcare than the lower-middle income countries. It is necessary to increase the research investment of the lower-middle income countries and cooperation with the developed countries. Extensive collaboration were presented between the developed countries, while collaboration between low-and middle-income countries and developed countries is limited. Collaborative efforts between developed and developing countries in this field require strengthening. Enhanced international cooperation is pivotal for advancing knowledge sharing, technical exchanges, and further development in the field. Radboud University, alongside other leading institutions such as the University of Pennsylvania, maintains robust collaborative ties, positioning it as a significant academic hub. Professor Bastiaan R. Bloem has a notable publication and citation record in PD care research, influencing research directions and focal points in the field through exemplary practices and innovative methods.

Further analysis identified hotspots and emerging trends in this domain. Keyword and research hotspot analyses indicate “Parkinson’s Disease”, “Quality of Life”, and “Dementia” as central themes, highlighting their significance in both research and clinical practice. Notably, the rising prominence of the keyword “Alzheimer’s Disease” suggests a growing focus in PD research on comorbidities with neurodegenerative disorders and their effects on treatment and care strategies. These findings underscore the evolving nature of the field and the need for continued exploration of comorbidities and their impact on PD care.

The co-authorship network analysis reveals a strong pattern of collaboration among researchers, particularly evident in the connectivity of core authors (25). This finding highlights the importance of strengthening scientific capabilities through cross-institutional and cross-national collaborations for future scientific advancements. With the discovery of biomarkers in PD, the application of gene-editing technologies, the development of novel medications, the exploration of new therapeutic approaches, as well as the integration of telemedicine and digital health monitoring tools, the field is experiencing rapid growth. However, this study also highlights the global disparities in resource distribution, as low-resource countries face challenges in accessing care resources, professional training, and research infrastructure. This emphasizes the need to address these imbalances through enhanced international cooperation and global sharing of scientific outputs.

This study has certain limitations. Firstly, our data is exclusively sourced from the WOSCC, which May not be comprehensive enough. Additionally, we only screened English articles, thus excluding articles published in other languages.

## 5 Conclusion

The study conducted a comprehensive bibliometric analysis of research literature in the field of Parkinson’s disease (PD) care, revealing an increasing trend in research and care attention resulting from global population aging and shifts in lifestyle. Research priorities are focused on quality of life and dementia-related issues associated with PD. However, activities are predominantly concentrated in developed countries, highlighting a deficiency in international collaboration. Future research should emphasize inclusivity and cooperation, especially in developing nations with uneven resource distribution and limited care capabilities, to promote balanced global advancement in PD care and research.

## Data Availability

The original contributions presented in the study are included in the article/Supplementary material, further inquiries can be directed to the corresponding authors.
