# Supplementary material for: Mapping the research of nursing in Parkinson’s disease: a bibliometric and quantitative analysis
Source: Front Neurol. 2024 Sep 25;15:1412158. doi: 10.3389/fneur.2024.1412158 (PMC11461253; doi:10.3389/fneur.2024.1412158)
Supplement: Supplementary file 1 [file Table_1.DOCX]

**Table S1: Top 5 Authors Ranked by Publication and Citations**

| Author | Number of Publications | Citation Count | Average Citation Count |
| --- | --- | --- | --- |
| Bastiaan R. Bloem | 76 | 2867 | 37.7 |
| Marten Munneke | 42 | 1539 | 36.6 |
| K. Ray Chaudhuri | 37 | 1338 | 36.2 |
| Angelo Antonini | 29 | 906 | 31.2 |
| Per Odin | 24 | 1538 | 64.1 |

**Table S2: Top 10 Countries and Institutions**

| Rank | Country | Publication Count | Centrality | Rank | Institution | Publication Count |
| --- | --- | --- | --- | --- | --- | --- |
| 1 | USA | 1057 | 384 | 1 | Radboud Universiteit Nijmegen | 99 |
| 2 | England | 400 | 384 | 2 | University of Pennsylvania | 81 |
| 3 | Germany | 317 | 302 | 3 | University of Toronto | 75 |
| 4 | Canada | 212 | 248 | 4 | King's College London | 69 |
| 5 | Netherlands | 205 | 231 | 5 | University of Rochester | 59 |
| 6 | Italy | 181 | 249 | 6 | University College London | 58 |
| 7 | Spain | 158 | 208 | 7 | University of Florida | 48 |
| 8 | France | 129 | 142 | 8 | Rush University | 44 |
| 9 | Australia | 128 | 103 | 9 | University of California, San Francisco | 44 |
| 10 | Sweden | 105 | 137 | 10 | Kings Coll Hosp London | 37 |

**Table S3: Top 10 Most Cited Journals**

| Rank | Journals | Publication Count | JCR | IF |
| --- | --- | --- | --- | --- |
| 1 | *Movement Disorders* | 234 | Q1 | 8.6 |
| 2 | *Parkinsonism & Related Disorders* | 138 | Q2 | 4.1 |
| 3 | *Journal of Neurology and Psychiatry* | 85 | Q1 | 8.2 |
| 4 | *Neurology* | 81 | Q1 | 9.9 |
| 5 | *Journal of Neurology* | 47 | Q2 | 6 |
| 6 | *Frontiers in Neurology* | 46 | Q3 | 3.4 |
| 7 | *Parkinson's Disease* | 36 | Q2 | 3.2 |
| 8 | *Journal of the American Geriatrics Society* | 21 | Q1 | 6.3 |
| 9 | *Journal of Neurology, Neurosurgery, and Psychiatry* | 20 | Q1 | 11 |
| 10 | *The Lancet Neurology* | 17 | Q1 | 48 |

**Table S4: Top 10 Most Co-Cited Journals**

| Rank | Cited Journals | Co-Cited Frequency | | JCR | IF |
| --- | --- | --- | --- | --- | --- |
| 1 | *Movement Disorders* | | 9947 | Q1 | 8.6 |
| 2 | *Neurology* | | 6816 | Q1 | 9.9 |
| 3 | *Parkinsonism & Related Disorders* | | 3825 | Q2 | 4.1 |
| 4 | *Journal of Neurology and Psychiatry* | | 2930 | Q1 | 8.2 |
| 5 | *Archives of Neurology* | | 1716 | / | / |
| 6 | *The Lancet Neurology* | | 1632 | Q1 | 48 |
| 7 | *New England Journal of Medicine* | | 1478 | Q1 | 158.5 |
| 8 | *Journal of Neurology* | | 1472 | Q2 | 6 |
| 9 | *Annals of Neurology* | | 1419 | Q1 | 11.2 |
| 10 | *Brain* | | 1342 | Q1 | 14.5 |

**Table S5. The top 5 Co-cited References**

| Reference | Co-cited Count | Authors |
| --- | --- | --- |
| Parkinsonism: onset, progression, and mortality | 331 | Margaret M. Hoehn |
| Accuracy of clinical diagnosis of idiopathic Parkinson's disease: a clinico-pathological study of 100 cases | 200 | Andrew J Hughes |
| Longer versus shorter daily constraint-induced movement therapy of chronic hemiparesis: an exploratory study | 186 | Annette Sterr |
| Projected number of people with Parkinson disease in the most populous nations, 2005 through 2030 | 140 | E. Ray Dorsey |
| Predictors of nursing home placement in Parkinson's disease: a population‐based, prospective study | 109 | Dag Aarsland |

**Table S6: Top 10 High-Frequency Keywords in PD and Nursing Research**

| Keywords | Frequency | TLS |
| --- | --- | --- |
| parkinson's disease | 1595 | 1378 |
| quality-of-life | 532 | 858 |
| dementia | 333 | 461 |
| prevalence | 317 | 435 |
| people | 263 | 364 |
| depression | 232 | 404 |
| symptoms | 207 | 374 |
| nonmotor symptoms | 162 | 364 |
| levodopa | 162 | 302 |
| risk | 161 | 290 |
